# Ulcerative and pyogranulomatous pododermatitis due to *Pseudomonas luteola* infection in a domestic ferret (*Mustela putorius furo*): a case report with literature review of this emerging zoonotic disease in ferrets

**DOI:** 10.1007/s11259-024-10464-3

**Published:** 2024-07-23

**Authors:** Jacobo Giner, María Eugenia Lebrero, Diego López-Sahuquillo, Sergio Villanueva-Saz, Carles Juan-Sallés, Teresa Navarro, Antonio Fernández, Diana Marteles, Álex Gómez

**Affiliations:** 1 Menescalia Veterinary Clinic, Ismael Merlo, 5, 46020 Valencia, Spain; 2 Clinical Immunology Laboratory, Veterinary Faculty, University of Zaragoza, Zaragoza, Spain; 3 Department of Animal Pathology, Veterinary Faculty, University of Zaragoza, 50013 Zaragoza, Spain; 4 Instituto Agroalimentario de Aragón-IA2 (Universidad de Zaragoza-CITA), Zaragoza, Spain; 5 Noah’s Path, Arquitecto Santiago Pérez Aracil 30 Bajo (Centro Veterinario), 03203 Elche, Spain

**Keywords:** Ferret, Pododermatitis, *Pseudomonas luteola*, Spain

## Abstract

*Pseudomonas luteola (P.luteola), *formerly called* Chryseomonas luteola*, is a strict aerobic gram-negative bacillus, 0.8 to 1.0 µm wide and 1.5 to 2.5 µm long, considered an opportunistic pathogen found ubiquitously in humid environments, both in soil and water. It sporadically causes disease in animals and immunosuppressed humans or those subjected to invasive procedures such us peritoneal dialysis or catheterization. In ferrets, this infection was first described in Spain in 2012 and since then, cases have appeared occasionally in Finland, Austria, Australia, France, the United States and also in Spain. This pathogen is considered an emerging zoonotic disease in ferrets, causing respiratory disease, panniculitis, and abscesses due to pyogranulomatous or suppurative inflammation predominantly of the pleura, lung, mediastinum, panniculus or salivary glands, frequently with lethal consequences. The clinical case of a ferret, infected by *Pseudomona luteola,* presenting with ulcerative suppurative pododermatitis and ipsilateral popliteal purulent lymphadenitis, is described. Together with a complete resolution of the clinical case by means of a non-invasive medical management likely due to the rapid detection, identification, and treatment of the infection.

## Introduction

*Pseudomonas luteola*, formerly known as *Chryseomonas luteola*, is a Gram-negative, aerobic, rod-shaped bacteria, 0,8–1 µm wide and 1,5–2,5 µm long with a non-staining capsule. The normal habitat of this bacterium remains unknown, although it is frequently found in damp environments, found in soil, and water (Rahav et al. 1995; Chihab et al. 2004)*. P. luteola* is composed by a polysaccharide capsule with a multitrichous polar flagella that has been associated with cadmium and cobalt ion absorption. The bacteria forms yellow-orange pigmentated colonies (Ozdemir et al. 2005).

*P. luteola* is rarely a cause of disease in humans, but it has been found in medical implants, peritoneal dialysis catheters, in immunosuppressed patients and as a nosocomial infection in surgical procedures (Chihab et al. 2004; Barry 2021). *P. luteola* has also been described as a causative agent of endophthalmitis, endocarditis, peritonitis, septicemia and leg ulcers in patients with sickle cell disease (Tsakris et al. 2022; Casalta et al. 2005; Uy et al. 2007; Su et al. 2014; Bayhan et al. 2015).

In the veterinary literature, *P. luteola* infections that cause mortality have been reported in farmed rainbow trout (Altinok et al. 2007), none the less, it has also been found as a part of the normal gastrointestinal flora of healthy zebrafish (*Danio rerio*) (Cantas et al. 2012). This pathogen rarely causes disease in mammals, but it has been documented in cats (Milliron et al. 2021) causing pyogranulomatous panniculitis (Milliron et al. 2021) and ferrets (Schmidt et al. 2019).

This infection is considered an emergent disease in ferrets in Europe and presents with abscesses, panniculitis or respiratory disease (Baum et al. 2015; Martínez et al. 2012). This case report describes the isolation of *P. luteola* from a ferret with an ulcerative and pyogranulomatous pododermatitis, considered an atypical clinical presentation in this species; the role of this pathogen as an emerging zoonotic agent in ferrets is reviewed.

## Material and methods

### Case history

**Fig. 1:**
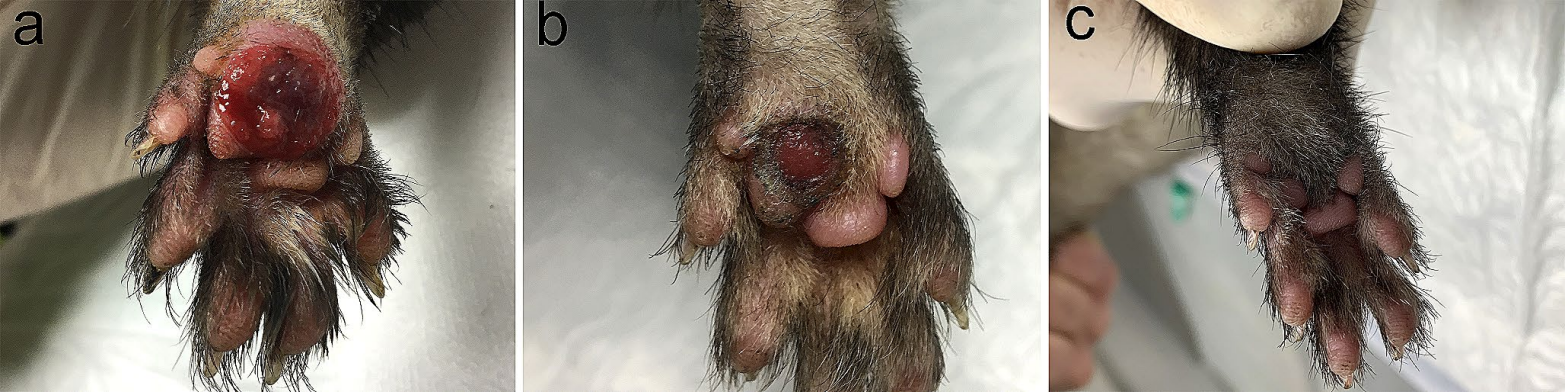
Clinical progression of the *P. luteola* associated pododermatitis. **a**). Severe, ulcerative and edematous pododermatitis previous to treatment; **b**). Improvement of pododermatitis after treatment; **c**). Complete resolution 1 month after completion of treatment

An 8-month-old intact male ferret (*Mustela puturius furo*) with a body weight of 1750 g was referred to the veterinary service for evaluation of an injury in the plantar aspect of the right hind limb, and the presence of lethargy and anorexia during the last 24 hours (Fig. 1a).

On clinical record, the ferret was fed a diet consisting of 70% raw food and 30% premium dry cat food. It was vaccinated against canine distemper receiving two doses of the vaccine with a one-month interval between them. The patient had an indoor lifestyle with outdoor access during weekends. The owners associated the wound with a field trip 6 days before. Physical examination was unremarkable other than pyrexia (40.5 ºC) and a painful, ulcerative and edematous lesion of 1 cm in diameter observed on the palmer aspect of the right paw, together with a firm and enlarged popliteal lymph node (1.5 cm × 1,75 cm) with no evidence of other lymph node enlargement. All other physiological parameters were within normal range, the ferret had a body condition score of 3/5, and cardiac and respiratory auscultation within normal limits. Two mls of blood were collected aseptically by jugular venipuncture for comprihensive blood tests including a blood count and a serum biochemical profile (Idexx, Westbrook, USA).

An incisional full-thickness biopsy of the ulcerative skin lesion was taken for histological examination. The ferret was premedicated with midazolam 0.2 mg/kg subcutaneously and butorphanol 0.2 mg/kg intramuscularly. Anesthesia was induced with alfaxalone 5 mg/kg administered intravenously, followed by tracheal intubation (2.0 mm) and maintenance with sevoflurane.

**Fig. 2:**
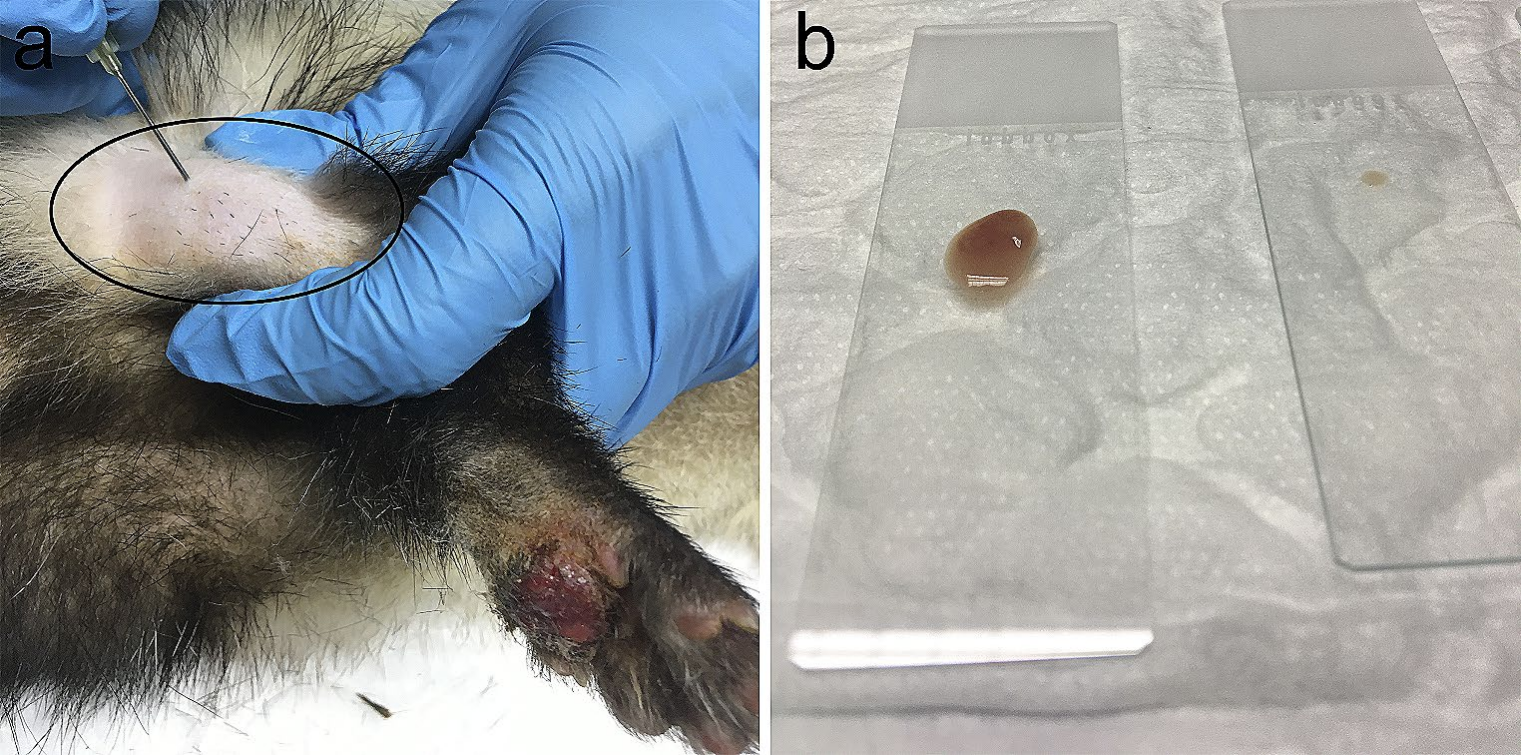
Fine needle aspiration (FNA) from the affected right popliteal lymph node. **a**). Popliteal lymph node enlargement; **b**). The sample was ejected on the slide for Diff-Quick staining and cytological examination

Additionally, various samples were taken by fine needle aspiration (FNA) of the affected right popliteal lymph node for cytological examination (Fig. 2). A Diff-Quick staining and bacterial culture was employed (isolation and antibiotic sensitivity testing was also undertaken). The aspirate was cultured on Blood agar (agar, 15 g/L, meat extract 10 g/L, peptone 10 g/L and sodium chloride 5 g/L, Merck), Chocolate Agar (146,093; Merck) and MacConkey agar (agar 12.0 g/L, bile salts 5.0 g/L, lactose 10.0 g/L, neutral red 0.075 g/L, peptone 20.0 g/L and sodium chloride 5.0 g/L, Merck). Two plates were incubated aerobically at 37 °C, and the other pairs of plates were incubated in anaerobic conditions at 37 °C. Plates were observed for bacterial growth after 16–20 h of cultivation. In cases of no or slow bacterial growth at first inspection, additional observations were performed at 24 h intervals. To isolate the types of growth colonies the MALDI-TOF mass spectrometry (VITEK MS, bioMérieux) and Knowledge Base database (version 3.0) was utilized. Finally, an antibiogram was performed. In addition, an incisional surgical biopsy of the dermal lesion was also obtained for histopathology tissue processing. This sample was fixed in 10% neutral-buffered formalin, embedded in paraffin, and 4 μm-thick sections were stained with hematoxylin and eosin (HE).

## Results

### Clinicopathological findings

Complete blood count, serum biochemistry, and serum protein electrophoresis revealed neutrophilia (7.56 (0.62–3.30 K/µL)), monocytosis (1,08 (0.18–0.90 K/µL)) and hyperglobulinemia (3.30 (1.80–3.10 g/dL)). While awaiting the results from cytology, bacterial culture and the biopsy, an empirical treatment was administered consisting of a combined antibiotic therapy of enrofloxacin (5 mg/kg/12 h) and amoxicillin-clavulanic acid (15 mg/kg/12 h), and meloxicam (0,1 mg/kg/24 h). With this treatment, the pododermatitis was partially resolved (Fig. 1b).

### Cytological examination

Cytological evaluation of a sample taken by FNA and stained with Diff-Quick revealed purulent lymphadenitis with the presence of a high number of markedly degenerative neutrophils, a few macrophages and the presence of low number of intracellular bacteria.

### Histopathological examination

**Fig. 3:**
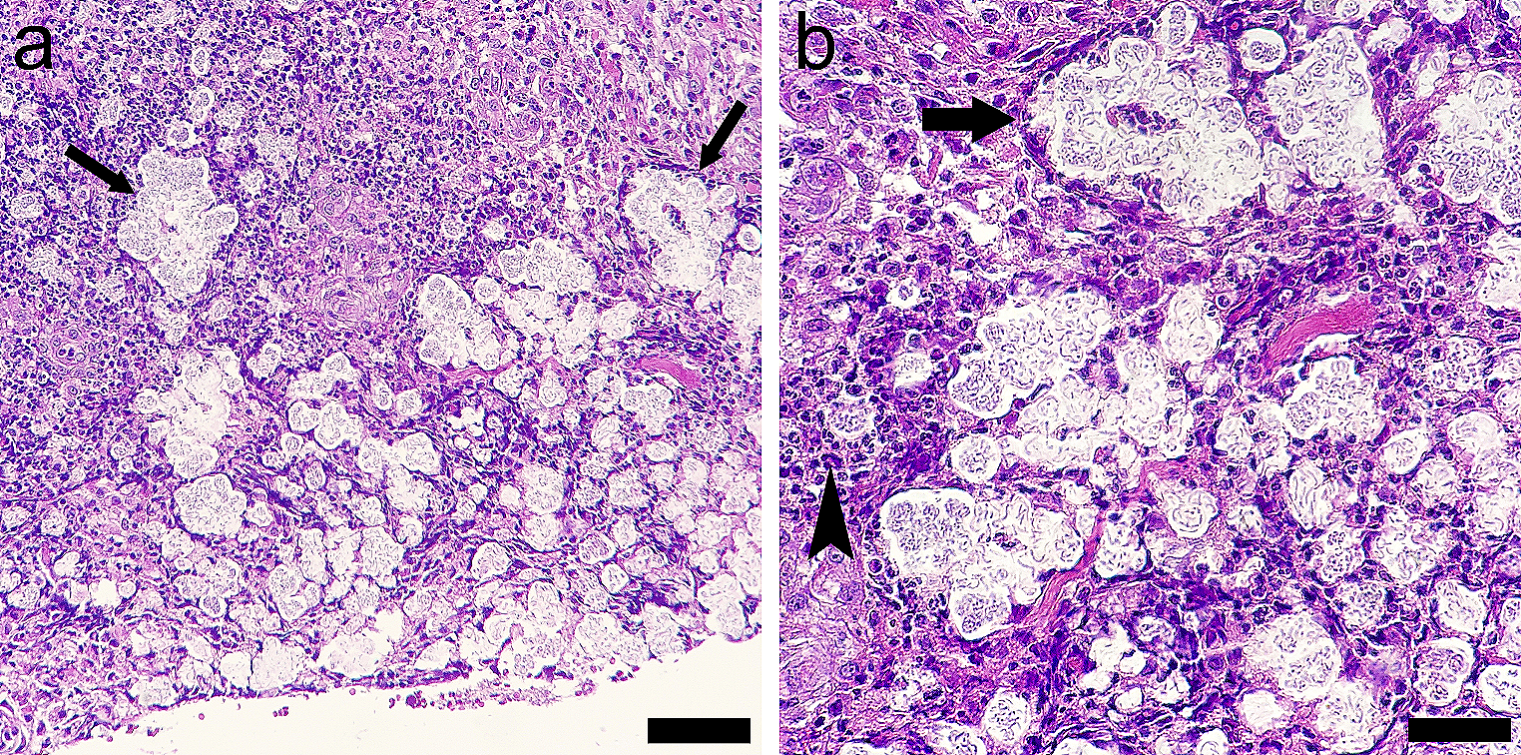
Histopathological analysis from sampled pododermatitis. **a**). Complete ulceration and colonies of cocobacilli and serpentine bacilli with a clear halo infiltrating the superficial and deep dermis (arrows). HE, Bar: 1000 μm.; **b**). Colonies (arrows) surrounded by neutrophils and macrophages (arrowhead). HE, Bar: 100 μm

Histopathological evaluation of the skin biopsy revealed an acute diffuse suppurative pododermatitis with ulceration and intralesional bacteria including coccobacilli and serpentine bacilli surrounded by a clear halo (Fig. 3).

### Bacterial culture and MALDI-TOF mass spectrometry

A single type of colony was isolated and identified as *P. luteola* using MALDI-TOF mass spectrometry. The antibiogram revealed that the *P. luteola* isolate was susceptible to ampicillin, amoxicillin-clavulanic acid, imipenem, meropenem, ciprofloxacin, enrofloxacin, marbofloxacin, pradofloxacin, gentamicin, tobramycin, doxycycline, cotrimoxazole, amikacin and tetracycline. *P. luteola* was resistant to cefalexin, cefuroxime, chloramphenicol and cefpodoxime. Finally, intermediate susceptibility was detected to trimethoprim-sulfamethoxazole and cefotaxime. Since the antibiogram revealed sensitivity to the antibiotics previously administered to the patient, a different antibiotic treatment was unnecessary. During the follow-up, the antibiotic therapy was continued for one month resulting in a complete resolution of pododermatitis and lymph node enlargement (Fig. 1c).

## Discussion

*P. luteola* is considered an emerging zoonotic disease in ferrets causing respiratory signs, abscesses or pyogranulomatous panniculitis (Wyre 2020). Although there has been an increase of case descriptions, involving *P. luteola,* in ferrets in recent years*,* there are no publications to date describing pododermatitis. The first clinical descriptions of *P. luteola* infection in ferrets were reported in Spain (Martínez et al. 2012). Later, additional clinical cases were documented in Australia (Coignet 2012), France (Coignet 2012), Finland and Austria (Baum et al. 2015), and the United States (Schmidt et al. 2019). In general, the most common clinical presentations of this infection in ferrets include thoracic or peritoneal lesions, with respiratory clinical signs or peritonitis, followed by the presence of abscesses in different tissue locations (Wyre 2020). There was no description of pododermatitis caused by this pathogen in ferrets.

The route of infection and mode of transmission of *P. luteola* in ferrets have not been determined. In previous reports with respiratory clinical signs, abscesses and subcutaneous edema, there has been no evidence of nosocomial infections or wound contamination. In the ferret of this report, infection might have been associated to a field trip with the owners 6 days before presentation. Since this bacterium is ubiquitous in damp environments, found in both soil and water (Rahav et al. 1995), an abrasion or penetrating trauma to the footpad with secondary bacterial inoculation from an environmental source is possible but cannot be confirmed.

Cytology and histopathology are necessary to focus the diagnosis of this bacterial disease. Microscopic findings such a purulent or pyogranulomatous inflammation with intralesional pleomorphic bacteria including serpentine bacilli surrounded by a clear halo should suggest *P. luteola* infection as an aetiology. However, to confirm the diagnosis of *P.luteola,* bacterial isolation and identification are required. In these cases, pure cultures of yellow-pigmented colonies involving gram-negative rods are the common findings in Blood and Mac-Conkey agars (Martínez et al. 2012). In the present report, *P. luteola* was isolated in Blood Agar, Agar MacConkey and also in Chocolate Agar, and it was identified by mass spectrophotometry MALDI-TOF.

*Pseudomonas* spp. are one of the major pathogens causing healthcare-associated infections. Its capacity of adaptation, dissemination, intrinsic resistance to antimicrobials and ability to gain new mechanisms through mobile genetic elements, make the treatment of infections by this microorganism a challenge for the clinician. For example, *P. aeruginosa*, presents a reduced permeability in the external membrane, due to the expression of efflux pumps, and an inducible AmpC-type cephalosporinase or benzyl-penicillinase (Morita et al. 2014). In addition, *P. aeruginosa* is able to acquire new resistance determinants by horizontal transfer in the form of cassettes located in integrons, and in turn located in transposons or plasmids. *P. aeruginosa* produces beta-lactamases, but presents resistance to beta-lactam antibiotics (including extended spectrum) and carbapenem antibiotics (Oliver 2017). Resistance of *P. luteola* to first and second-generation cephalosporins, tetracycline, ampicillin and trimethoprim-sulfamethoxazole has been documented (Rahav et al. 1995; Yousefi et al. 2014). In other case reports in ferrets, treatment was based on antibiogram results and included amoxicillin, with clavulanic acid, amikacin or enrofloxacin (Schmidt et al. 2019). In this report, resistance to cephalexin and cefuroxime (first and second-generation of cephalosporins), cefovecin (third-generation cephalosporin) and chloramphenicol was found and *P. luteola* was sensitive to fluoroquinolones and other beta-lactam antibiotics, as previously described (Schmidt et al. 2019). Probably, *P. luteola* possesses different mechanisms of responses and resistance compared to *P. aeruginosa.*

This is the first case report of pododermatitis caused by *P. luteola* in a ferret. Complete resolution with medical treatment alone was possible. This success may be related to the promptness of the detection, identification and treatment. Consequently, *P. luteola* should be included in the dermatological differential diagnosis of pododermatitis in ferrets. In the majority of cases described in the literature, the diagnosis was made “post mortem” and in the few cases in which satisfactory treatment could be established, it was based on combined medical and surgical treatments. In the patient described in this case report, complete resolution was obtained with medical treatment alone, possibly due to the rapid detection, identification, and treatment of the infection. This case reinforces that *P. luteola* is more frequently sensitive to non-cephalosporin beta-lactams, and amoxicillin with clavulanic acid should be recommended as a first option in cases without an antibiogram.

## Data Availability

No datasets were generated or analysed during the current study.

## References

[CR1] Altinok I, Balta F, Capkin E, Kayis S (2007) Disease of rainbow trout caused by *Pseudomonas luteola*. Aquaculture 273:393–397

[CR2] Barry M (2021) *Pseudomonas luteola* bacteremia in newly diagnosed systemic lupus erythematosus. Case Rep Infect Dis 14:4051378 10.1155/2021/4051378 PMC8382557 34434585

[CR3] Baum B, Richter B, Reifinger M, Klang A, Finnberg C, Loncaric I, Spergser J, Eisenberg T, Künzel F, Preis S, Pantchev N, Rütgen B, Guija de Arespacochaga A, Hewicker-Trautwein M (2015) Pyogranulomatous panniculitis in ferrets (*Mustela putorius furo*) with intralesional demonstration of *Pseudomonas luteola*. J Comp Pathol 152:114–118 25728813 10.1016/j.jcpa.2015.01.003

[CR4] Bayhan GI, Senel S, Tanir G, Ozkan S (2015) Bacteremia caused by *Pseudomonas luteola* in pediatric patients. Jpn J Infect Dis 68:50–54 25420649 10.7883/yoken.JJID.2014.051

[CR5] Cantas L, Sørby JR, Aleström P, Sørum H (2012) Culturable gut microbiota diversity in zebrafish. Zebrafish 9:26–37 22428747 10.1089/zeb.2011.0712 PMC3308716

[CR6] Casalta JP, Fournier PE, Habib G, Riberi A, Raoult D (2005) Prosthetic valve endocarditis caused by Pseudomonas luteola. BMC Infect Dis 5:82 16221303 10.1186/1471-2334-5-82 PMC1274313

[CR7] Chihab W, Alaoui AS, Amar M (2004) *Chryseomonas luteola* identified as the source of serious infections in a Moroccan University Hospital. J Clin Microbiol 42:1837–1839 15071064 10.1128/JCM.42.4.1837-1839.2004 PMC387548

[CR8] Coignet S (2012) Etude retrospective des infections a Pseudomonas luteola chez le furet. http://theses.vet-alfort.fr/telecharger.php?id=1893. Accessed 25 September 2023

[CR9] Martínez J, Martorell J, Abarca ML, Olvera A, Ramis A, Woods L, Cheville N, Juan-Sallés C, Moya A, Riera A, Soto S (2012) Pyogranulomatous pleuropneumonia and mediastinitis in ferrets (*Mustela putorius furo*) associated with *Pseudomonas luteola* infection. J Comp Pathol 146:4–10 21601873 10.1016/j.jcpa.2011.03.014 PMC7094560

[CR10] Milliron SM, Seyler ZG, Myers AN, Rodrigues Hoffmann A, Hnot M, Wiener DJ (2021) Pyogranulomatous panniculitis in a domestic cat associated with *Pseudomonas luteola* infection. Vet Dermatol 32:83-e15 32991013 10.1111/vde.12893

[CR11] Morita Y, Tomida J, Kawamura Y (2014) Responses of *Pseudomonas aeruginosa* to antimicrobials. Front Microbiol 4(Jan):1–8 10.3389/fmicb.2013.00422 PMC3884212 24409175

[CR12] Oliver A (2017) Epidemiology and carbapenem resistance mechanisms in *Pseudomonas aeruginosa:* Role of high-risk clones in multidrug resistance. Enferm Infecc Microbiol Clin 35(3):137–138 28161004 10.1016/j.eimc.2016.11.006

[CR13] Ozdemir G, Ceyhan N, Manav E (2005) Utilization of an exopolysaccharide produced by *Chryseomonas luteola* TEM05 in alginate beads for adsorption of cadmium and cobalt ions. Bioresour Technol 96:1677–1682 16023570 10.1016/j.biortech.2004.12.031

[CR14] Rahav G, Simhon A, Mattan Y, Moses AE, Sacks T (1995) Infections with *Chryseomonas luteola* (CDC group Ve-1) and flavimonas oryzihabitans (CDC group Ve-2). Medicine 74:83–88 7891546 10.1097/00005792-199503000-00003

[CR15] Schmidt L, Doss G, Hawkins S, Blake C, Baumwart R, Kalish R, Rizzi T, Dreyfus J, Brandao J (2019) Cranial cervical abscessation and sialadenitis due to *Pseudomonas luteola* in two domestic ferrets (*Mustela putorius furo*) J Exot Pet Med 31:120–126

[CR16] Su SY, Chao CM, Lai CC (2014) Peritoneal dialysis peritonitis caused by *Pseudomonas luteola*. Perit Dial Int 34:138–139 24525608 10.3747/pdi.2012.00265 PMC3923710

[CR17] Tsakris A, Hassapopoulou H, Skoura L, Pournaras S, Douboyas J (2022) Leg ulcer due to *Pseudomanas luteola* in a patient with sickle cell disease. Diagn Microbiol Infect Dis 42:141–143 10.1016/s0732-8893(01)00336-4 11858911

[CR18] Uy HS, Leuenberger EU, de Guzman BB, Natividad FF (2007) Chronic, postoperative Pseudomonas luteola endophthalmitis. Ocul Immunol Inflamm 15:359–361 17763136 10.1080/09273940701396697

[CR19] Wyre NR (2020) Emerging zoonotic diseases in ferrets. Vet Clin North Am Exot Anim Pract 23:299–308 32327037 10.1016/j.cvex.2020.01.012

[CR20] Yousefi F, Shoja S, Honarvar N (2014) Empyema caused by Pseudomonas luteola: a case report. Jundishapur J Microbiol 7:e10923 25368791 10.5812/jjm.10923 PMC4216571

